# Supplementary material for: Characterization and Involvement of Exosomes Originating from Chikungunya Virus-Infected Epithelial Cells in the Transmission of Infectious Viral Elements
Source: Int J Mol Sci. 2022 Oct 11;23(20):12117. doi: 10.3390/ijms232012117 (PMC9603488; doi:10.3390/ijms232012117)

Supplementary Figure S1: **The workflow illustration of the EVs isolation from the cell-cultured medium used in this study.** (**A**) A schematic representation of total exosome isolation using the Total Exosomes Isolation (from cell culture media) kit was indicated. (**B**) Invitrogen™ Dynabeads™ Exosome- CD63 isolation kit was used for purification assay of exosome subpopulations.

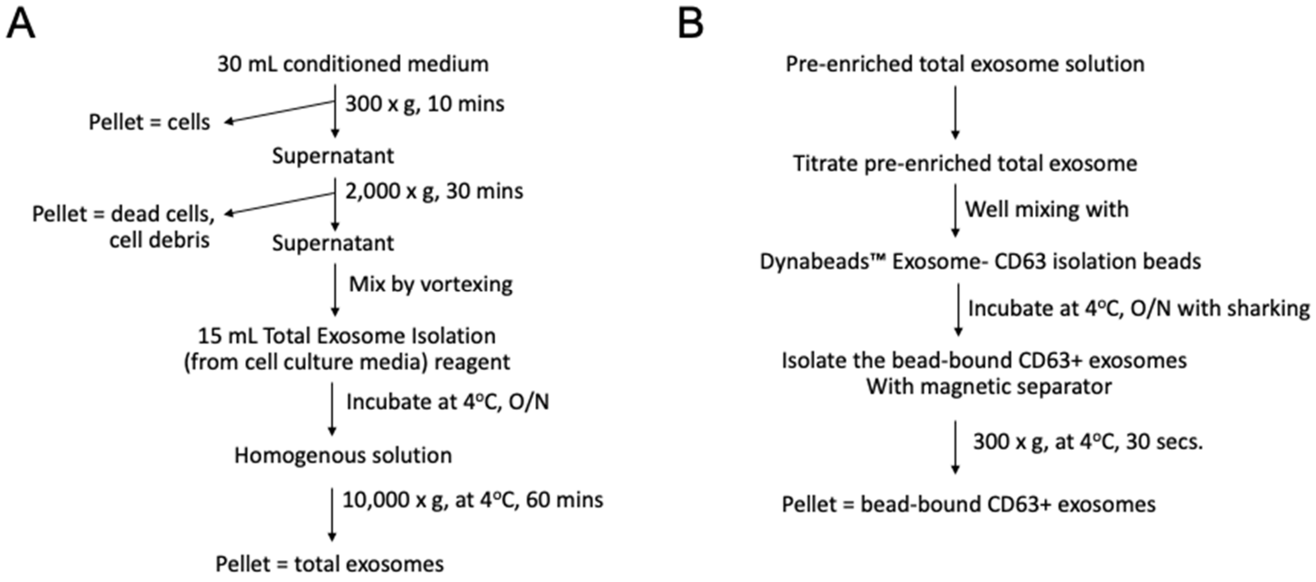

Supplement: Supplementary file 1 [file ijms-23-12117-s001.zip › 4. Supplementary Figures.pdf]
