# Supplementary material for: Characterization and Involvement of Exosomes Originating from Chikungunya Virus-Infected Epithelial Cells in the Transmission of Infectious Viral Elements
Source: Int J Mol Sci. 2022 Oct 11;23(20):12117. doi: 10.3390/ijms232012117 (PMC9603488; doi:10.3390/ijms232012117)
Supplement: Supplementary file 1 [file ijms-23-12117-s001.zip › 5. Supplementary Table.pdf]

Supplementary Table S1: **Oligonucleotide used in this study.**

Targets	Sequence (5′--> 3′)	Amplicon size	Refs.
**For CHIKV genome**			
5′UTR-Capsid	F: CACGTAGCCTACCAGTTTCTTA<br>R: CGCTTGAAGGCCAATTTGGCC	8,005 bp	Modified from Stapleford et al, 2016 [36]
Capsid-3′UTR	F: GCAGAGAGAGAATGTGCATG<br>R: AACATCTCCTACGTCCCTATGG	4,114 bp	
**For CHIKV mRNA loads**			
E1	F: GCTCCGCGTCCTTTACC<br>R: GCGGGTAGTCCATGTTGTAA	176 bp	Designed in this study
β-actin	F: GACTTCGAGCAGGAGATGGC<br>R: ATTCCATGCCCAGGAAGCAA	148 bp	Designed in this study
